# Bad Air

**Published:** 1883-11

**Authors:** 


### BAD AIR.

WHEN a person has remained for an hour or more in a crowded and poorly ventilated room or railroad car, the system is already contaminated to a greater or less extent by breathing air, vitiated by exhalations from the lungs, bodies and clothing of the occupants. The immediate effect of these poisons is to debilitate, to lower vitality, and to impair the natural power of the system to resist disease. Hence it is that persons who are attacked by inflammatory diseases, as pneumonia or rheumatism, can generally trace the beginning of the disease to a chill felt on coming out of a crowded room into the cold or damp air, wearing perhaps thin shoes and insufficient clothing. If these facts were generally understood and acted upon, thousands of lives might be saved every year.

It is a well-known fact that men who "camp out," sleeping on the ground at all seasons of the year, seldom have pneumonia, and that rheumatism, with them, comes, as a rule, only from unwarrantable imprudences.

There are two facts that should be learned by every person capable of appreciating them, and they should never be lost sight of for a moment. One is that exhalations from the lungs—the breath—is a deadly poison, containing the products of combustion in the form of carbonic acid gas, and if a person were compelled to reinhale it unmixed with the oxygen of the air, it would prove as destructive to life as the fumes of charcoal. This is an enemy that is always present, in force, in assemblies of people, and only a constant and free infusion of fresh air prevents it from doing mischief that would be immediately apparent.

The other fact is that pure air is the antidote to this poison. The oxygen of the air is the greatest of all purifiers. Rapid streams of water that pass through large cities, receiving the sewage, become pure again through the action of the air after running a few miles. Air is the best of all "blood purifiers." Combined with vigorous exercise to make it effective, it will cure any curable case of consumption.

Biting the Finger Nails.—This is a very common and a very unpleasant habit. If one has not the firmness to stop it, the habit may be radically cured by having the ends of the fingers cut off, or the teeth extracted. It will not be necessary to do both.

